# Supplementary material for: Earlier ice loss accelerates lake warming in the Northern Hemisphere
Source: Nat Commun. 2022 Sep 2;13:5156. doi: 10.1038/s41467-022-32830-y (PMC9440048; doi:10.1038/s41467-022-32830-y)

Supplementary Information for

Earlier ice loss accelerates lake warming in the Northern Hemisphere

Xinyu Li[1], Shushi Peng[1*], Yi Xi[1], R. Iestyn Woolway[2], Gang Liu[1]

[1]Sino-French Institute for Earth System Science, College of Urban and Environmental Sciences, and Key Laboratory for Earth Surface Processes, Peking University, Beijing, China

[2]School of Ocean Sciences, Bangor University, Menai Bridge, Anglesey, UK

* Correspondence to Shushi Peng (speng@pku.edu.cn)

Contents of this file

Supplementary Tables 1 to 4

Supplementary Figures 1 to 20

Supplementary Table 1. Correlation coefficients (R-squared) between the trend in lake surface water temperature during the ice-off month ($LSWT_{IOFF}$) and the ice-on month ($LSWT_{ION}$), the ratios of $LSWT_{IOFF}$ to lake warming trend during the open-water season (R_{IOFF} and R_{ION}), the sensitivity of lake surface temperature to changes in ice-off dates (S_{IOFF}), and ice-on dates (S_{ION}), and lake latitude, lake size, and lake elevation for individual lakes in ARC-Lake and lake grid cells in ERA5. Statistically significant correlation coefficients at the 99.9% ($p < 0.001$) level are denoted by one asterisk (*), with R-squared in parentheses.

	Individual lakes in ARC-Lake (n = 963)			Lake grid cells in ERA5 (n = 109,405)	
	Latitude	Lake size	Lake elevation	Latitude	Lake elevation
Trend in $LSWT_{IOFF}$	0.06(0.00)	-0.01(0.00)	0.09(0.01)	0.30(0.09)*	-0.24(0.06)*
Trend in $LSWT_{ION}$	0.16(0.03)*	-0.02(0.00)	-0.08(0.01)	0.37(0.14)*	-0.26(0.07)*
R_{IOFF}	-0.03(0.00)	-0.05(0.00)	0.03(0.00)	0.07(0.00)*	-0.14(0.02)*
R_{ION}	-0.02(0.00)	-0.02(0.00)	-0.04(0.00)	0.16(0.02)*	-0.19(0.03)*
S_{IOFF}	-0.12(0.01)	0.05(0.00)	0.07(0.00)	-0.24(0.06)*	0.12(0.01)*
S_{ION}	0.11(0.01)	0.07(0.00)	-0.04(0.00)	0.20(0.04)*	-0.09(0.01)*

Supplementary Table 2. Correlation coefficients (R-squared) between the trend in $LSWT_{IOFF}$ / $LSWT_{ION}$ and the trend in climate variables (air temperature (Tair), downward short-wave radiation (SWdown), and downward long-wave radiation (LWdown)) during the ice-off / ice-on month and lake depth. All correlation coefficients shown in the table are statistically significant at the 99.9% ($p < 0.001$) level, with R-squared in parentheses.

	Trend in Tair	Trend in SWdown	Trend in LWdown	Trend in ice-off / ice-on date	Depth	Number of ice-free days during the ice-off / ice-on month
Trend in $LSWT_{IOFF}$	0.25 (0.06)	-0.01 (0.00)	0.32 (0.10)	-0.61 (0.37)	-0.49 (0.24)	0.37 (0.13)
Trend in $LSWT_{ION}$	0.45 (0.20)	-0.12 (0.01)	0.42 (0.17)	0.63 (0.39)	-0.14 (0.02)	-0.39 (0.15)

Supplementary Table 3. Trend in climate variables (Tair, SWdown, and LWdown) and lake surface water temperature (LSWT) during the open-water season, ice-off month, and ice-on month. All results are presented as mean ± standard deviation.

	Trend in Tair (°C $year^{-1}$)	Trend in SWdown (W m^{-2} $year^{-1}$)	Trend in LWdown (W m^{-2} $year^{-1}$)	Trend in LSWT (°C $year^{-1}$)
Open-water season	0.04 ± 0.02	0.07 ± 0.20	0.14 ± 0.08	0.03 ± 0.02
Ice-off month	0.04 ± 0.03	0.06 ± 0.32	0.13 ± 0.14	0.04 ± 0.04
Ice-on month	0.05 ± 0.04	-0.04 ± 0.13	0.17 ± 0.18	0.02 ± 0.02

Supplementary Table 4. Correlation coefficients (R-squared) between the trend in ice-off date and the trend in climate variables (Tair, SWdown, and LWdown) during the ice-off month and one to three months after the ice-off month and lake depth. All correlation coefficients shown in the table are statistically significant at the 99.9% ($p < 0.001$) level, with R-squared in parentheses.

Month after the ice-off month	Trend in Tair	Trend in SWdown	Trend in LWdown	Depth	Trend in ice-off date
0	0.25(0.06)	-0.01(0.00)	0.32(0.10)	-0.49(0.24)	-0.61(0.37)
1	0.52(0.27)	0.25(0.06)	0.21(0.04)	-0.06(0.00)	-0.49(0.24)
2	0.61(0.38)	0.11(0.01)	0.38(0.14)	0.15(0.02)	-0.34(0.11)
3	0.03(0.00)	0.33(0.11)	0.12(0.01)	0.02(0.00)	-0.18(0.03)

Supplementary Figure 1. Relationships between lake ice cover and lake size, lake elevation, and lake latitude for individual lakes in ARC-Lake and lake grid cells in ERA5. **a-b**, Relationship of ice cover and lake size (**a**) and lake latitude (**b**) for lakes in ARC-Lake. **c**, Relationship of ice cover and lake latitude for lakes in ERA5. The colors in **b-c** indicate lake elevation. **d-f**, Spatial patterns of lake size (**d**), lake elevation (**e**) for lakes in ARC-Lake, and lake elevation for lakes in ERA5 (**f**).

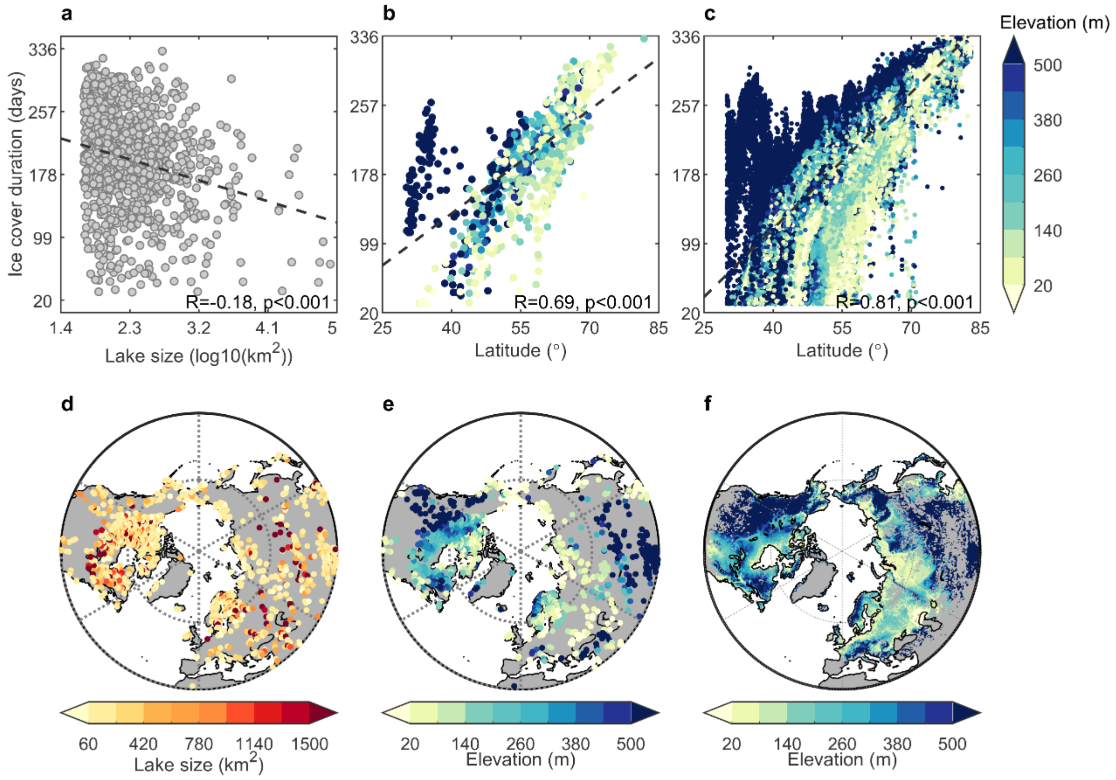

Supplementary Figure 2. Excess lake warming in the ice-off or ice-on month derived from ARC-Lake during the period 1995–2012. **a**-**b**, Spatial pattern of mean ice-off month (**a**), ice-on month (**b**). **c**-**d**, Spatial pattern of ratios of the trend in LSWT in the ice-off month ($LSWT_{IOFF}$) or LSWT in the ice-on month ($LSWT_{ION}$) to the trend in LSWT during the open-water period (R_{IOFF} (**c**) and R_{ION} (**d**)). The inset in **a**-**d** shows the probability density function (PDF) from Europe (orange), North America (pink), and Asia (blue), respectively.

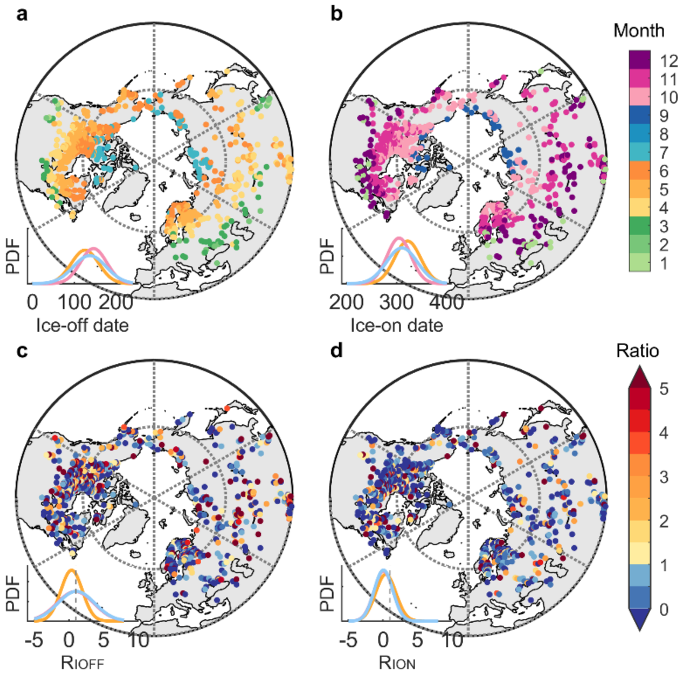

Supplementary Figure 3. Explanation of RIOFF and RION by three climate variables. **a-d**, Spatial patterns of RIOFF (**a**), trends in air temperature (Tair) (**b**), downward short-wave radiation (SWdown) (**c**), and downward long-wave radiation (LWdown) (**d**) in the ice-off month. **e-g**, Relationships between RIOFF and the trend in Tair (**e**), RIOFF and the trend in SWdown (**f**), and RIOFF and the trend in LWdown (**g**). **h-k**, Spatial patterns of RION (**h**), trends in Tair (**i**), SWdown (**j**), and LWdown (**k**) during the ice-on month. **l-n**, Relationship between RION and the trend in Tair (l), RION and the trend in SWdown (**m**), and RION and the trend in LWdown (**n**).

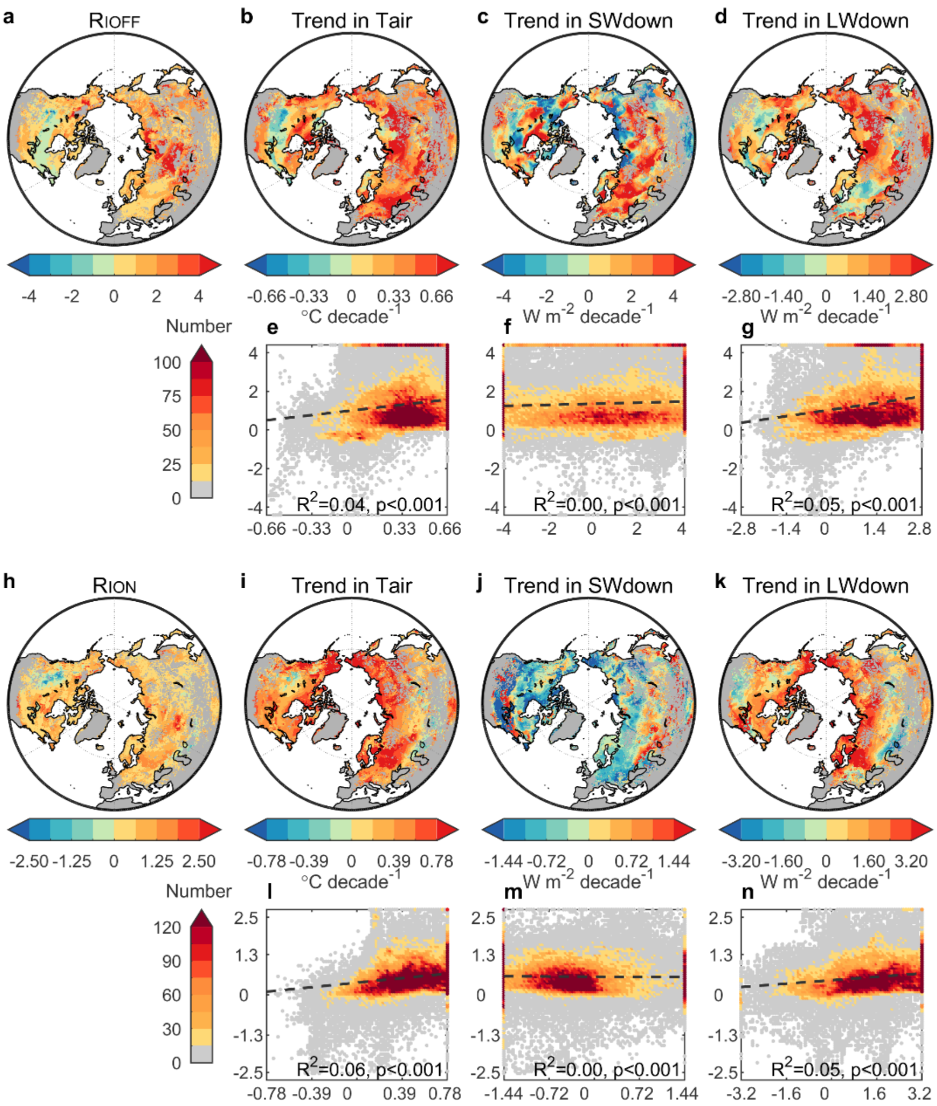

Supplementary Figure 4. Explanation for slight cooling of lakes in southern Canada. Spatial pattern of the trend in lake surface water temperature during the ice-off month ($LSWT_{IOF}$) (**a**), and the trend in ice-off date (**b**). Spatial pattern of the trend in air temperature (Tair) (**c**) and downward short-wave radiation (SWdown) (**e**) during the preceding month before the ice-off month, as well as during the ice-off month (**d**, **f**).

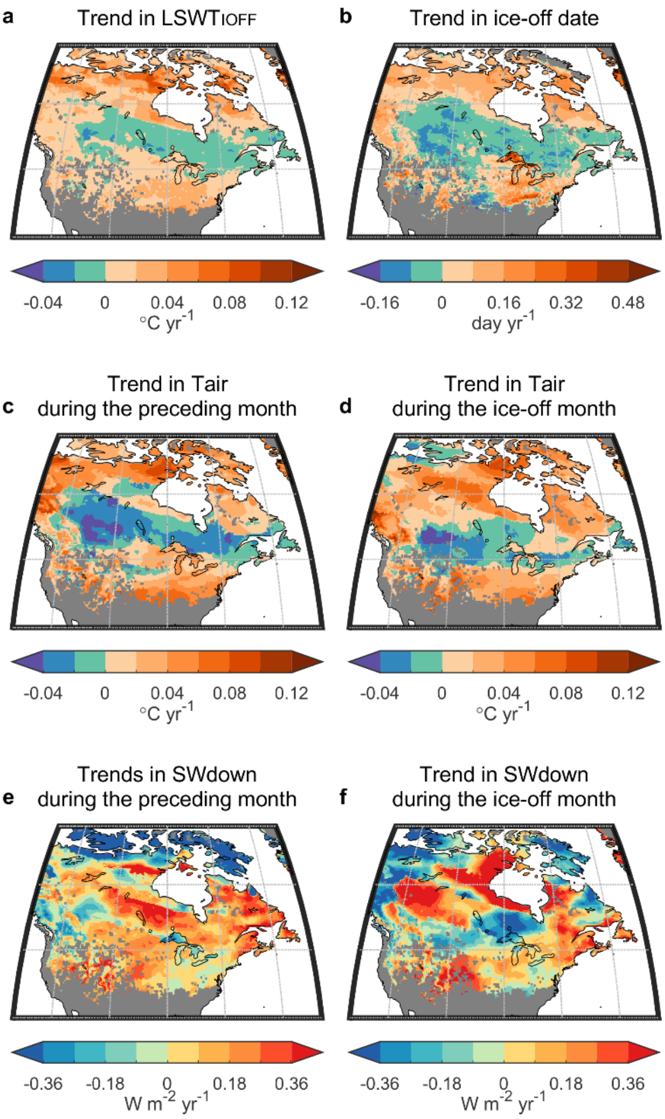

Supplementary Figure 5. Relationship between excess warming during the ice-off month or the ice-on month and extra incoming radiation due to changes of lake ice phenology. **a-d**, Spatial patterns of trend in $\text{LSWT}_{\text{IOFF}}$ (**a**), ice-off date (**b**), ΔE_{IOFF}^{SW} (**c**), and ΔE_{IOFF}^{LW} (**d**). **e-g**, Relationship of the trend in $\text{LSWT}_{\text{IOFF}}$ and trend in ice-off date (**e**), ΔE_{IOFF}^{SW} (**f**) and ΔE_{IOFF}^{LW} (**g**). Positive values of ΔE_{IOFF}^{SW} and ΔE_{IOFF}^{LW} indicate extra incoming radiation due to earlier ice-off date. **h-k**, Spatial patterns of trend in LSWT_{ION} (**h**), ice-off date (**l**), ΔE_{ION}^{SW} (**j**) and ΔE_{ION}^{LW} (**k**). **l-n**, Relationship of the trend in LSWT_{ION} and trend in ice-on date (**l**), ΔE_{ION}^{SW} (**m**) and ΔE_{ION}^{LW} (**n**). Positive values of ΔE_{ION}^{SW} and ΔE_{ION}^{LW} indicate extra incoming radiation due to later ice-on date. The colors in **e-g**, **l-n** indicate the number of lake grid cells.

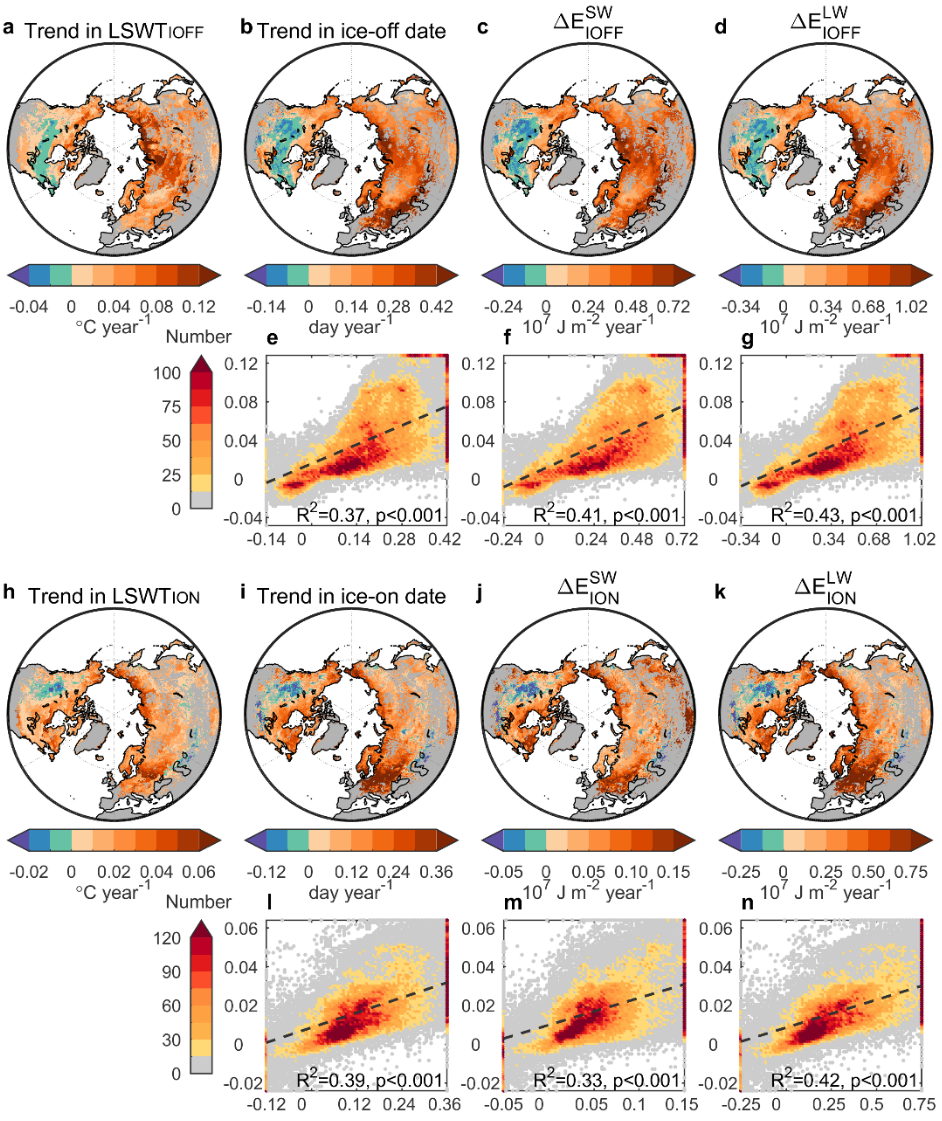

Supplementary Figure 6. Relationship between excess warming during the ice-off month or the ice-on month and extra incoming radiation due to changes of lake ice phenology, using lake surface temperature data from ARC-lake from 1995 to 2012. **a**-**d**, Spatial patterns of trend in $LSWT_{IOFF}$ (**a**), ice-off date (**b**), ΔE_{IOFF}^{SW} (**c**), and ΔE_{IOFF}^{LW} (**d**). **e**-**g**, Relationship of the trend in $LSWT_{IOFF}$ and trend in ice-off date (**e**), ΔE_{IOFF}^{SW} (**f**) and ΔE_{IOFF}^{LW} (**g**). Positive values of ΔE_{IOFF}^{SW} and ΔE_{IOFF}^{LW} indicate extra incoming radiation due to earlier ice-off date. **h**-**k**, Spatial patterns of trend in $LSWT_{ION}$ (**h**), ice-off date (**l**), ΔE_{ION}^{SW} (**j**) and ΔE_{ION}^{LW} (**k**). **l**-**n**, Relationship of the trend in $LSWT_{ION}$ and trend in ice-on date (**l**), ΔE_{ION}^{SW} (**m**) and ΔE_{ION}^{LW} (**n**). Positive values of ΔE_{ION}^{SW} and ΔE_{ION}^{LW} indicate extra incoming radiation due to later ice-on date. The colors in **e**-**g**, **l**-**n** indicate the number of lake grid cells.

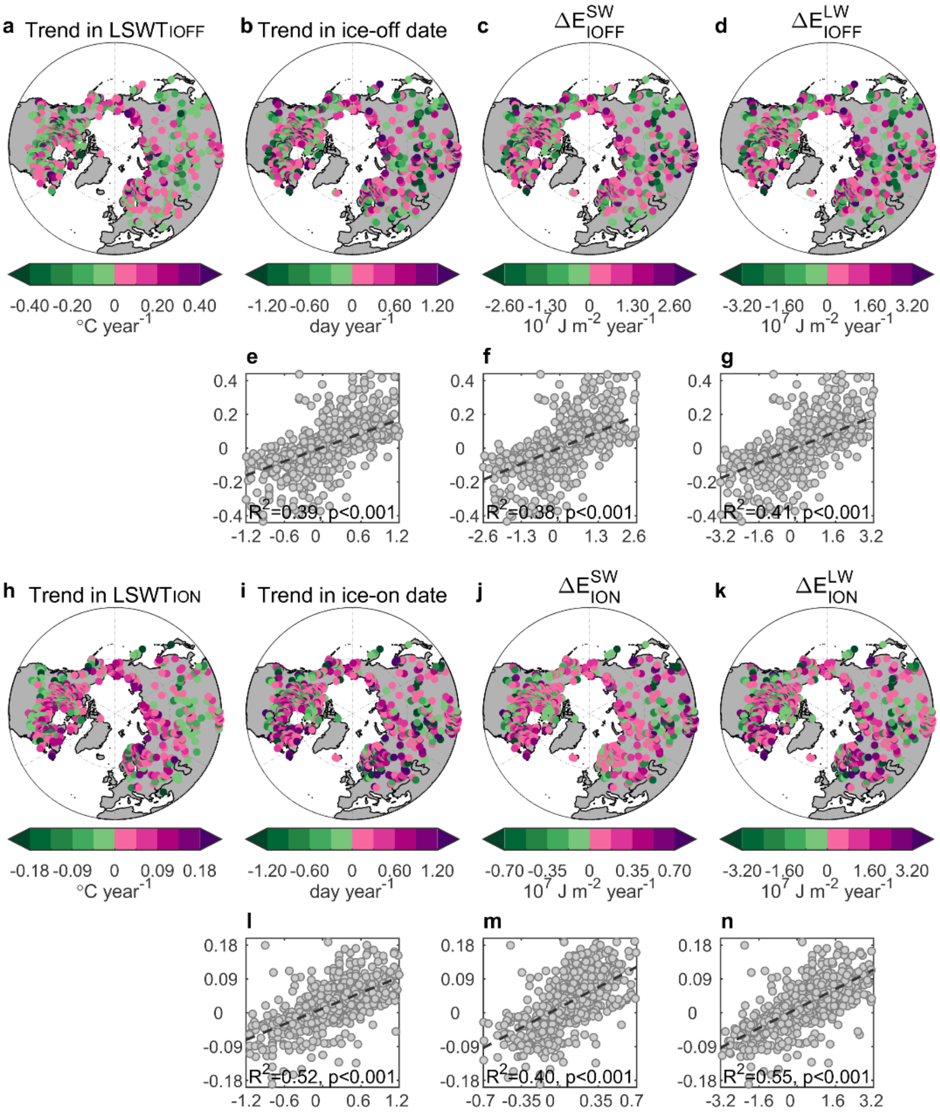
a Trend in LSWTIOFF
b Trend in ice-off date
c ΔE SW IOFF
d ΔE LW IOFF
-0.40 -0.20 0 0.20 0.40
°C year-1
-1.20 -0.60 0 0.60 1.20
day year-1
-2.60 -1.30 0 1.30 2.60
10^7 J m-2 year-1
-3.20 -1.60 0 1.60 3.20
10^7 J m-2 year-1
e
f
g
R2=0.39, p<0.001
R2=0.38, p<0.001
R2=0.41, p<0.001
h Trend in LSWTION
i Trend in ice-on date
j ΔE SW ION
k ΔE LW ION
-0.18 -0.09 0 0.09 0.18
°C year-1
-1.20 -0.60 0 0.60 1.20
day year-1
-0.70 -0.35 0 0.35 0.70
10^7 J m-2 year-1
-3.20 -1.60 0 1.60 3.20
10^7 J m-2 year-1
l
m
n
R2=0.52, p<0.001
R2=0.40, p<0.001
R2=0.55, p<0.001

Supplementary Figure 7. Lake ice phenology and stratification phenology across the Northern Hemisphere using lake temperature data from ERA5 during the period 1979–2020. Spatial patterns of mean ice-off month (**a**), mean onset month of stratification (**b**), trend in ice-off date (**c**), and trend in stratification onset (**d**).

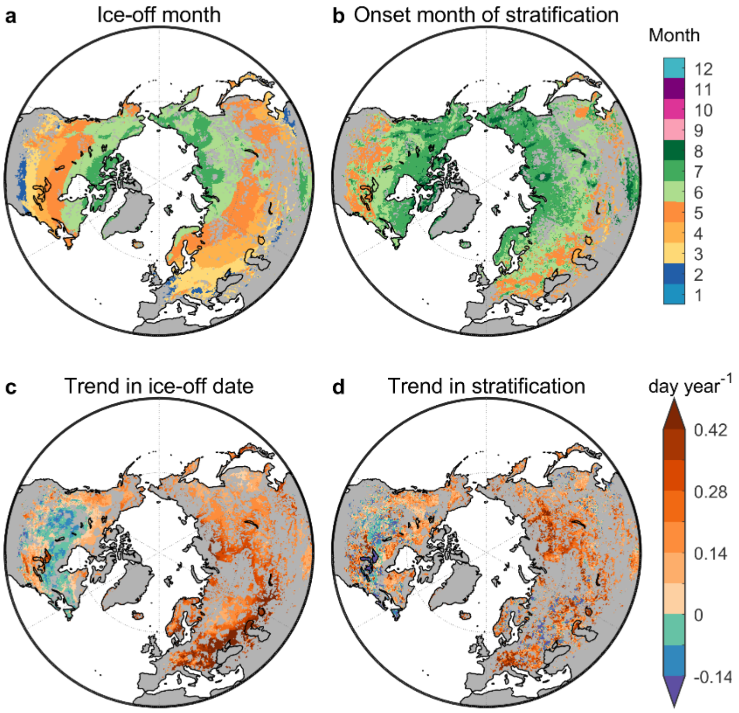

Supplementary Figure 8. Spatial patterns of the Tair (**a**), SWdown (**b**), LWdown (**c**), and sum of SWdown and LWdown (**d**) during the ice-off month, as well as during the ice-on month (**e-h**).

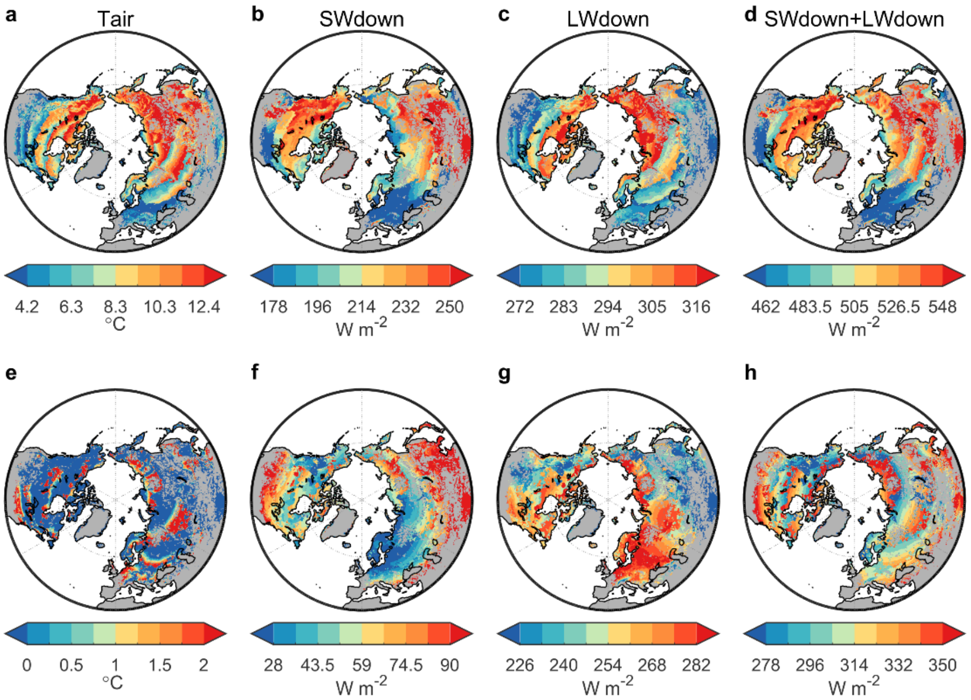

Supplementary Figure 9. Spatial patterns of the trends in annual lake surface temperature (**a**) and seasonal lake surface temperature in spring (March to May) (**b**), summer (June to August) (**c**), autumn (September to November) (**d**) from ERA5 during the period 1979–2020.

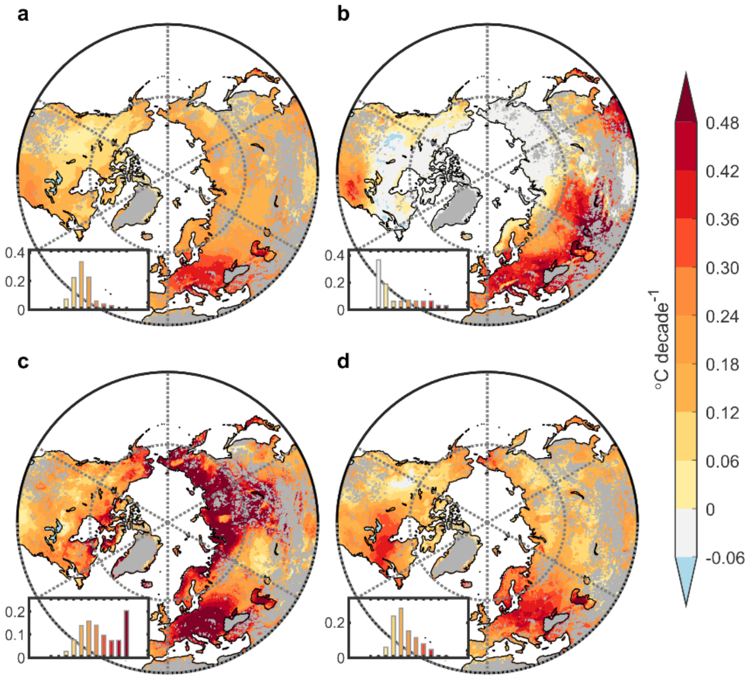

Supplementary Figure 10. Relationship between ice-off month and month with the maximum trend in lake surface water temperature (LSWT) in Europe (**a**), Asia (**b**), and North America (**c**). The color indicates the density of lake grid cells, and the darker color represents more lake grid cells.

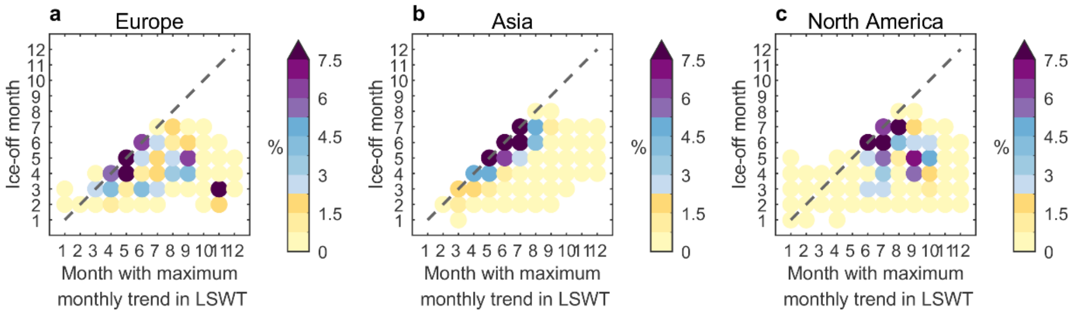

Supplementary Figure 11. Comparison of annual and seasonal lake warming rates from ERA5 and ARC-Lake during the period 1995-2012. **a**-**d**, Spatial patterns of trends in annual (**a**), spring (**b**), summer (**c**), and autumn (**d**) LSWT from ERA5. **e**-**f**, Spatial pattern of trends in annual (**e**), spring (**f**), summer (**g**), and autumn (**h**) LSWT from ARC-Lake. Trends in annual and seasonal mean lake surface temperature for each grid cell were estimated by least-squares linear regression. **i**-**l**, Annual (**i**) and seasonal (**j**-**l**) mean lake warming rates in Europe, Asia, and North America from two lake surface temperature products. Horizontal black lines indicate mean lake warming rates, and the top and bottom edges of boxes indicate the 75th and 25th percentiles of lake warming rates in each continent.

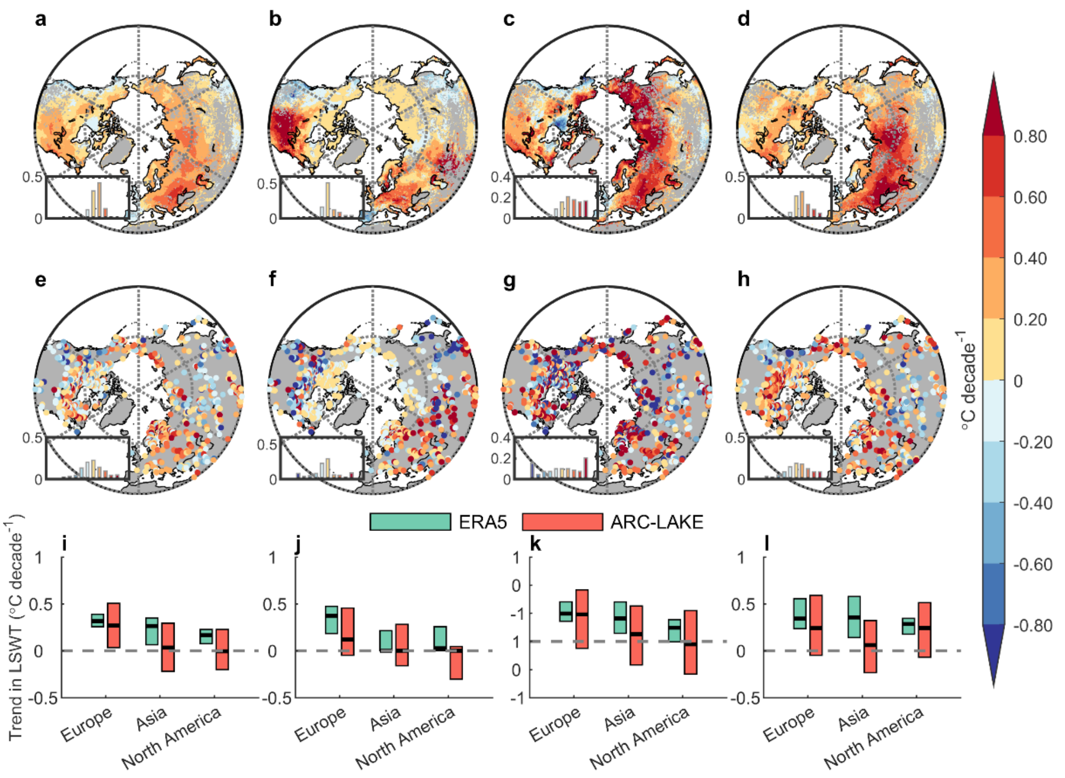

Supplementary Figure 12. Comparison of annual and seasonal lake warming rates from ERA5 and ESA-CCI during the period 2007-2019. **a-d**, Spatial patterns of trends in annual (**a**), spring (**b**), summer (**c**), and autumn (**d**) LSWT from ERA5. **e-f**, Spatial pattern of trends in annual (**e**), spring (**f**), summer (**g**), and autumn (**h**) LSWT from ESA-CCI. Trends in annual and seasonal mean lake surface temperature for each grid cell were estimated by least-squares linear regression. **i-l**, Annual (**i**) and seasonal (**j-l**) mean lake warming rates in Europe, Asia, and North America from two lake surface temperature products. Horizontal black lines indicate mean lake warming rates, and the top and bottom edges of boxes indicate the 75th and 25th percentiles of lake warming rates in each continent.

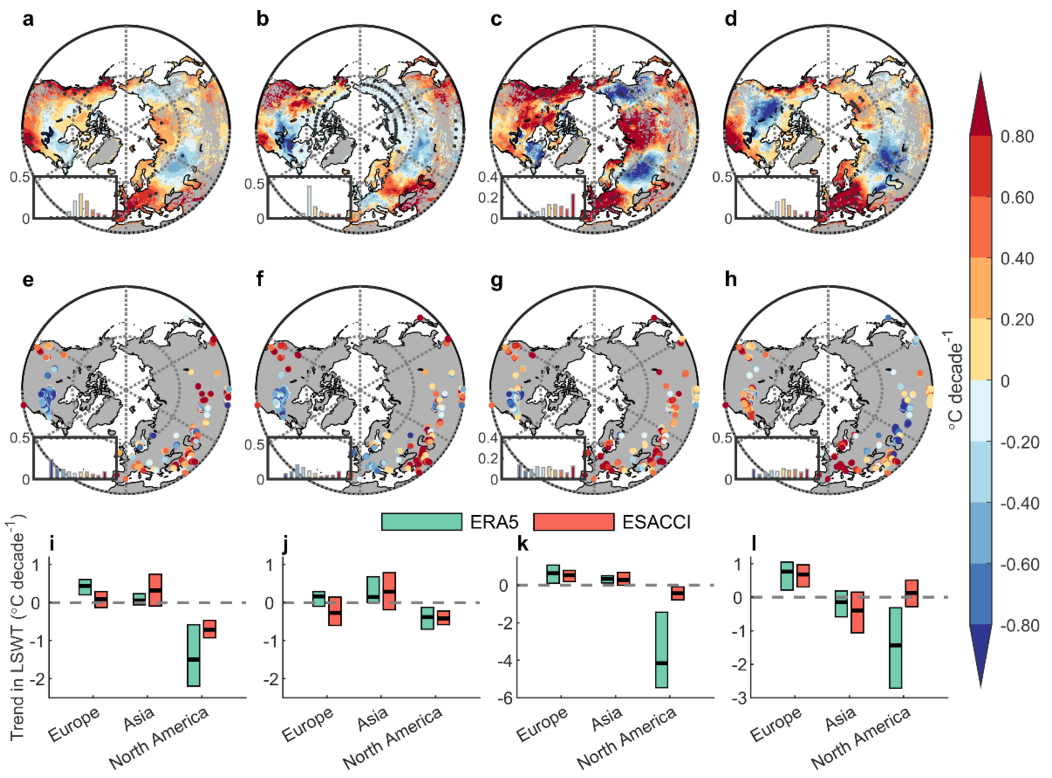

Supplementary Figure 13. Comparison of annual ice-off (**a**) and ice-on date (**b**) derived from AMSR-E/2 and ERA5 lake surface temperature from 2002 to 2015. Colors indicate the number of lake grid cells, and the redder color represents more lake grid cells. Dashed lines indicate 1:1 line.

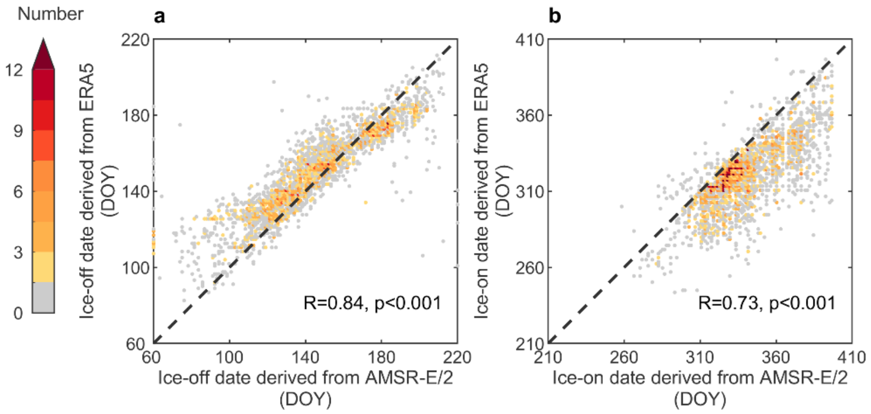

Supplementary Figure 14. Comparison of annual ice-off (**a**) and ice-on date (**b**) derived from ARC-Lake and AMSR-E/2 lake surface temperature from 2002 to 2012. Colors indicate the number of lake grid cells, and the redder color represents more lake grid cells. Dashed lines indicate 1:1 line.

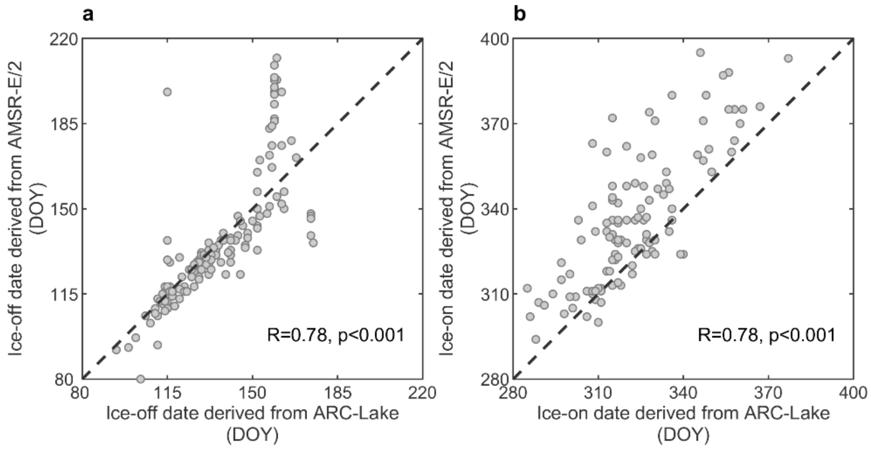

Supplementary Figure 15. Comparison of annual ice-off (**a**) and ice-on date (**b**) derived from ARC-Lake and ERA5 lake surface temperature from 1995 to 2012. Colors indicate the number of lake grid cells, and the redder color represents more lake grid cells. Dashed lines indicate 1:1 line.

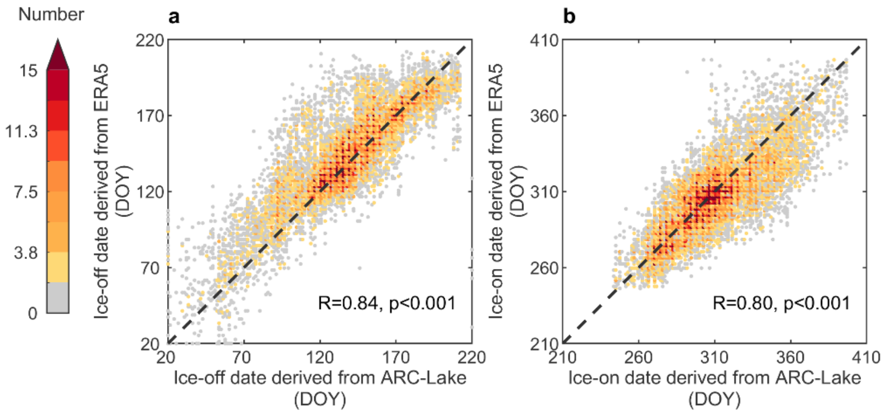

Supplementary Figure 16. Comparison of annual ice-off (**a**) and ice-on date (**b**) derived from LSWT in ERA5 and lake ice thickness in ERA5 from 1979 to 2020. Colors indicate the number of lake grid cells, and the redder color represents more lake grid cells. Dashed lines indicate 1:1 line.

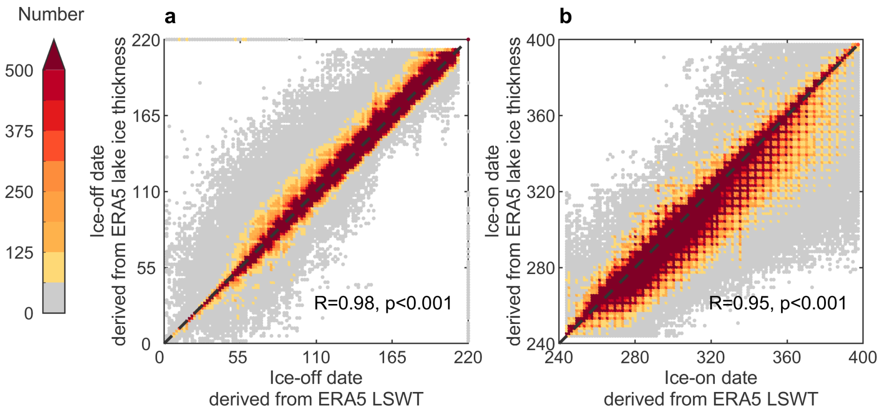

Supplementary Figure 17. Comparison of annual ice-off (**a**) and ice-on date (**b**) derived from AMSR-E/2 and ERA5 lake ice thickness from 2002 to 2015. Colors indicate the number of lake grid cells, and the redder color represents more lake grid cells. Dashed lines indicate 1:1 line.

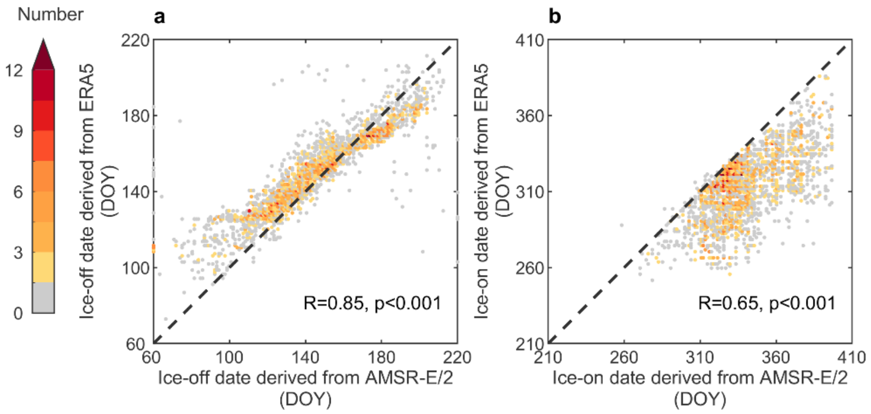

Supplementary Figure 18. Comparison of trend in annual lake ice phenology calculated using lake ice thickness and LSWT from ERA5. **a**-**b**, Spatial patterns of trend in annual ice-off dates calculated using lake ice thickness (**a**) and LSWT (**b**) from 1979 to 2020. **c**, Relationships between trend in ice-off dates derived from the two variables. **d**-**e**, Spatial patterns of trend in annual ice-on dates calculated using lake ice thickness (**d**) and LSWT (**e**) in ERA5 from 1979 to 2020. **f**, Relationships between trend in ice-off dates derived from the two variables. Dashed lines in (**e**) and (**f**) indicate 1:1 line.

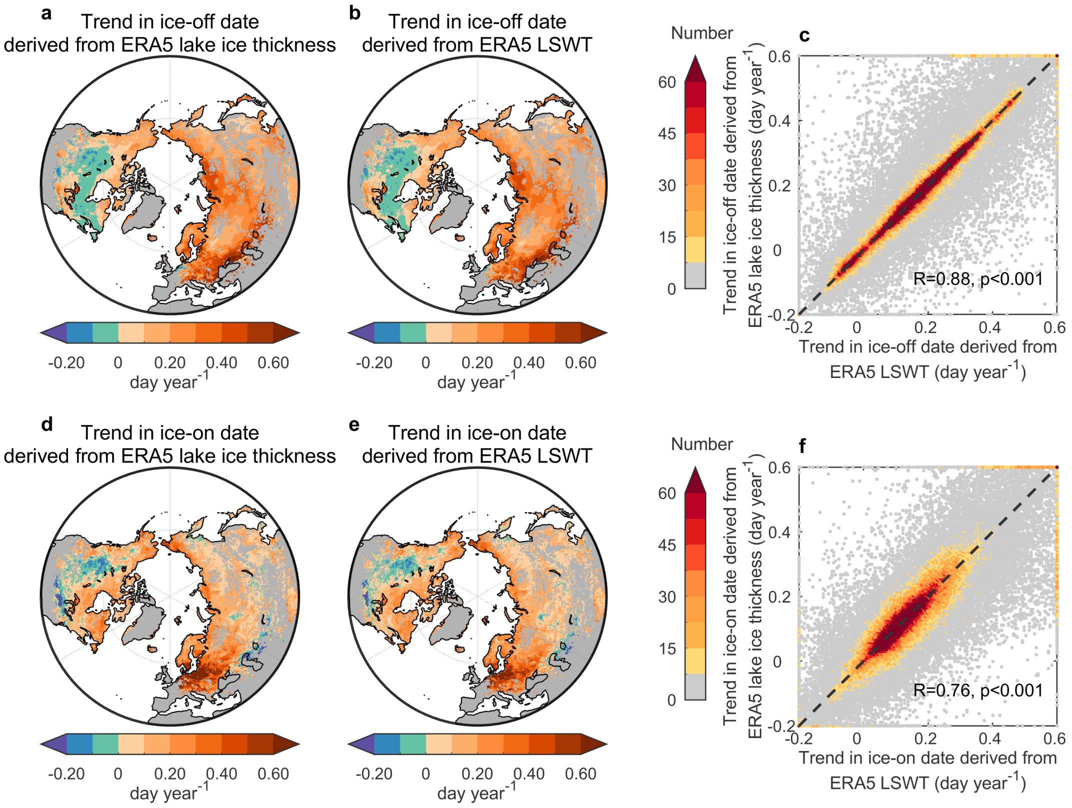

Supplementary Figure 19. Explanation for excess warming in the ice-off or ice-on month, but regarding the following month as the ice-off month when mean ice-off date is later than the 25th day of the month and regarding the previous month as the ice-on month when mean ice-on date is earlier than the 5th day of the month. **a**-**c**, Spatial pattern of the trend in $LSWT_{IOFF}$ (**a**), the trend in ice-off date (**b**), and lake depth (**c**). **d**-**e**, Relationship between the trend in $LSWT_{IOFF}$ (y-axis) and the trend in ice-off date (**d**) and lake depth (**e**) (x-axis). Positive values in (**b**) and the x-axis of (**d**) indicate earlier ice-off dates across lakes. **f**-**h**, Spatial pattern of the trends in $LSWT_{ION}$ (f), ice-on dates (**g**), and downward longwave radiation (LWdown) in the ice-on month (**h**). i-j, Relationship between the trend in $LSWT_{ION}$ (y-axis) and the trend in ice-on dates (**d**) and the trend in downward LWdown (**e**) in the ice-on month (x-axis). Positive values in (**g**) and the x-axis of (**i**) indicate later ice-on dates across lakes. The color of points in **d**-**e** and **i**-**j** shows the number of lakes.

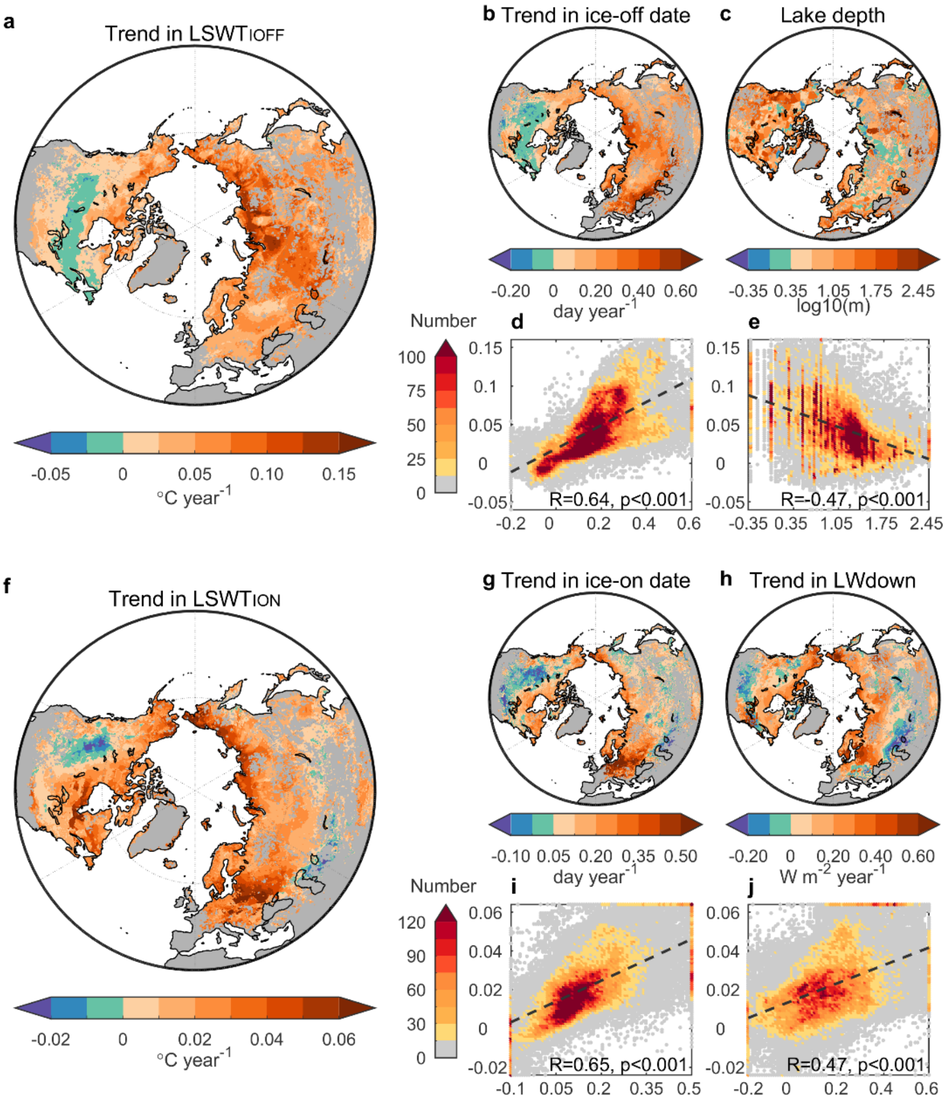

a
Trend in LSWTIOFF
-0.05 0 0.05 0.10 0.15
°C year-1
b Trend in ice-off date
-0.20 0 0.20 0.40 0.60
day year-1
c Lake depth
-0.35 0.35 1.05 1.75 2.45
log10(m)
Number
d
R=0.64, p<0.001
e
R=-0.47, p<0.001
f
Trend in LSWTION
-0.02 0 0.02 0.04 0.06
°C year-1
g Trend in ice-on date
-0.10 0.05 0.20 0.35 0.50
day year-1
h Trend in LWdown
-0.20 0 0.20 0.40 0.60
W m-2 year-1
Number
i
R=0.65, p<0.001
j
R=0.47, p<0.001

13

Supplementary Figure 20. Schematic of the lake vertical structure and temperature profile during complete mixing (**a**) and stratification (**b**).

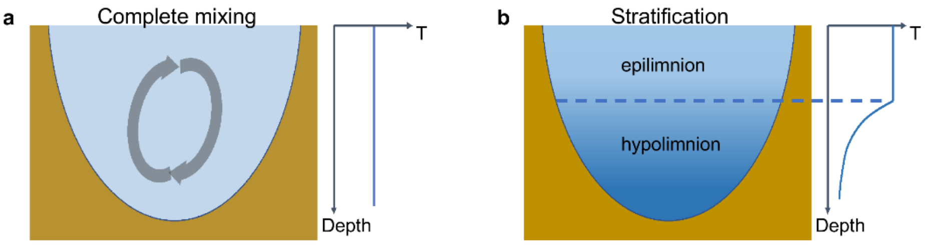

Supplement: Supplementary file 1 — Supplementary Information [file 41467_2022_32830_MOESM1_ESM.pdf]
